# Failed Sleeve Gastrectomy: Single Anastomosis Duodenoileal Bypass or Roux-en-Y Gastric Bypass? A Multicenter Cohort Study

**DOI:** 10.1007/s11695-018-3429-z

**Published:** 2018-07-31

**Authors:** Phillip J. Dijkhorst, Abel B. Boerboom, Ignace M. C. Janssen, Dingeman J. Swank, René M. J. Wiezer, Eric J. Hazebroek, Frits J. Berends, Edo O. Aarts

**Affiliations:** 1 Dutch Obesity Clinic, Huis ter Heide, Netherlands; 2 Department of Surgery, Rijnstate Hospital/Vitalys Clinics, Arnhem, Netherlands; 3 Department of Surgery, NOK-West/HMC and Groene Hart, The Hague and Gouda, Netherlands; 4 Department of Surgery, St. Antonius Hospital, Nieuwegein, Netherlands

**Keywords:** Morbid obesity, Bariatric surgery, Sleeve gastrectomy, SG, Gastric bypass, RYGB, duodenoileal bypass, SADI, Weight loss

## Abstract

**Background:**

Sleeve gastrectomy (SG) has become the most performed bariatric procedure to induce weight loss worldwide. Unfortunately, a significant portion of patients show insufficient weight loss or weight regain after a few years.

**Objective:**

To investigate the effectiveness of the single anastomosis duodenoileal (SADI) bypass versus the Roux-en-Y gastric bypass (RYGB) on health outcomes in morbid obese patients who had undergone SG previously, with up to 2 years of follow-up.

**Methods:**

From 2007 to 2017, 140 patients received revisional laparoscopic surgery after SG in four specialized Dutch bariatric hospitals. Data was analyzed retrospectively and included comparisons for indication of surgery, vitamin/mineral deficiencies, and complications; divided into short-, medium-term. To compare weight loss, linear regression and linear mixed models were used.

**Results:**

Conversion of a SG to SADI was performed in 66 patients and to RYGB in 74 patients. For patients in which additional weight loss was the main indication for surgery, SADI achieved 8.7%, 12.4%, and 19.4% more total body weight loss at 6, 12, and 24 months compared to RYGB (all *p* < .001). When a RYGB was indicated in case of gastroesophageal reflux or dysphagia, it greatly reduced complaints almost directly after surgery. Furthermore, a similar amount of complications and nutritional deficiencies was observed for both groups. There was no intra- or post-operative mortality.

**Conclusion:**

Conversion into a SADI resulted in significantly more weight loss while complications rates and nutritional deficiencies were similar and may therefore be considered the recommended operation for patients in which only additional weight loss is required.

## Introduction

The sleeve gastrectomy (SG), as derived from the first step of the duodenal switch procedure, has recently become the most performed bariatric procedure worldwide. It has especially gained popularity during the past decade because of its relative simplicity compared to, for example, the Roux-en-Y gastric bypass. In the short-term, the SG yields good results for weight loss and comorbidity resolution [1]. However, if weight loss is inadequate or patients experience weight regain, they are advised to undergo revisional surgery. This is especially apparent in those with a higher initial BMI before the SG [2, 3]. Other patients have satisfactory weight loss but suffer from functional complications such as severe gastroesophageal reflux disease (GERD) or dysphagia due to a stenosis. Perhaps more concerning are recent reports of patients developing Barrett esophagus as soon as 5 years post-SG, which theoretically increases the risk of esophageal carcinoma [4–7]. To compound matters, these patients do not benefit enough from the SG as a stand-alone procedure and are advised to undergo revisional surgery.

As the duodenal switch is technically demanding and associated with a high rate of perioperative morbidity [8, 9], other procedures for revisional surgery are needed. The question still remains which bariatric procedure should be performed as revisional surgery after a SG. Two available options include the single anastomosis duodenoileal (SADI) bypass and RYGB. The SADI has been introduced by Sanchez-Pernaute, A et al. (2007) as a simplification of the biliopancreatic diversion and duodenal switch [10]. It is suggested that weight loss results are similar to those obtained after the duodenal switch, but complications rates and nutritional deficiencies might be less frequent [11–13]. However, data available upon this matter are scarce, especially for revisional surgery. A second option is the RYGB, which has been used regularly for many years and has proven its effect in bariatric surgery as a safe and effective primary as well as revisional procedure [14–17]. A major advantage for GERD patients is that in a RYGB, the restrictive function of the pylorus is bypassed, which is why this operation is the best option to reverse GERD symptoms [18]. However, questions have been raised regarding failure rates following RYGB [19, 20].

To date, studies on SADI following SG reported on only small sample sizes. Furthermore, a comparison with the RYGB as a second step has never been made. The aim of this study is to investigate the effectiveness of the SADI versus the RYGB on health outcomes in morbid obese patients who have undergone SG with up to 2-year follow-up.

## Methods

### Patient Selection and Data Collection

Patients who underwent revisional bariatric surgery after SG to SADI or RYGB at one of four Dutch bariatric hospitals (of the five performing SADI in the Netherlands) from 2007 to 2017 were included in this study. These hospitals include the Haaglanden Medical center in The Hague, Groene Hart in Gouda, Rijnstate in Arnhem and St. Antonius in Nieuwegein. The institutional review board approved this retrospective study prior to data collection. The patients included in the study were divided into two groups. The first group consisted of patients that were operated on in order to improve weight loss after either weight regain or insufficient weight loss. The second group consisted of patients that were operated on because of a functional problem with the SG (e.a. stenosis, reflux, or fistula). This group was analyzed separately. These indications were determined after a multidisciplinary consultation. Inclusion criteria consisted of a prior SG, age 18–65 years, BMI of > 35 kg/m^2^, and all other criteria described in the European guidelines for bariatric surgery by Fried, M. [21]. Exclusion criteria were known malignancies, pregnancy, or conditions associated with poor compliance (psychiatric illness).

Data were collected retrospectively from medical records, supplemented with data and laboratory results collected during the lifestyle program that is provided by the Dutch Obesity Clinic.

### Surgical Procedures

All surgical procedures were started laparoscopically; however, three had to be converted to an open laparotomy, of which two were a SADI and one a RYGB.

#### SG

The SG was performed as a primary operation [22–24]. First, the greater curvature and angle of His were dissected to staple the gastric fundus and greater curvature parallel to a 40 French gastric bougie, which is inserted in the stomach through the esophagus. Stapling is started from a distance of 3–5 cm from the pylorus on the side of greater curvature side toward the angle of His. This results in a tube-like stomach with a volume of approximately 100 cc made from the lesser curvature only. Respectively, a black, a green, a gold, and up to three additional blue cartridges are used without buttressing material.

#### SADI

The SADI was exclusively performed as a secondary procedure after a SG. Following an evaluation of the abdominal cavity, the stomach was held upwards to identify the pylorus and dissect the duodenum 3 cm distal of the pylorus. From the ileocecal junction, the surgeon measured 250 cm counting with 5-cm intervals to mark the point for anastomosis. This part was pulled cranially to be anastomosed with the proximal duodenal stump using a stapler and/or V-loc sutures. Two of the participating centers recently changed the common channel measurement to 300 cm, leading to four SADI patients with a common channel of 300 cm.

#### RYGB

After SG, the RYGB was performed by creating a 30–50-ml pouch using a linear stapler by transecting the sleeve at the level of the cardia. A Roux limb with a length of 100 cm was attached to the gastric pouch using a linear stapler with a running suture. The biliopancreatic limb was on average 150 cm in length, measured with a hand-over-hand technique along the mesenteric border.

### Post-operative Management

After revisional surgery, patients started with clear liquids and ambulation on the day of operation. A thrombosis prophylaxis (Fraxiparine ®5700 IU [GlaxoSmithKline Inc., Mississauga, Ontario, Canada]) was administered once a day for 28 days. Multivitamins from Fit For Me (FFM, Rotterdam, The Netherlands) were advised to all patients after revisional surgery; SADI patients received FFM maximum and RYGB patients received FFM forte.

### Outcomes

The primary outcome was weight loss following revisional surgery, defined as percentage total body weight loss (%TBWL, weight loss in kilograms at a follow-up time point divided by weight in kilograms measured at secondary operation or at the time of SG). Weight was measured with light clothes on only and to compensate 1 kg was deducted of the measured weight in kilograms. For the second operation, weight was measured on the day of revisional surgery. Follow-up weight was measured at 1.5, 3, 6, 12, 18, and 24 months following revisional surgery and yearly thereafter. These measurements were performed by doctors or specialized bariatric nurses in one of the hospitals or at the Dutch Obesity Clinic.

Secondary outcomes were complications following revisional surgery and change in vitamin or mineral status. Complications were divided into short-term (< 30 days), medium-term (> 1, < 12 months), and long-term (> 12 months). Within these time frames, the complications included readmission to the hospital and reoperation.

Blood tests were performed before the second operation, multiple times during the first year after secondary surgery, and then annually. Patients were diagnosed with a deficiency if a specific value for the mineral or vitamin was under a lower limit. The lower limits used are those reported by the American Society for Metabolic and Bariatric Surgery Integrated Health Nutritional Guidelines (2016) [25]. The percentage of patients that were deficient was calculated by dividing the number of patients with a deficiency by the total number of patients that were available. If patients did not show up at follow-up for serum level analyses, they were excluded from this analysis from that time on.

### Statistical Analysis

**Fig. 1:**
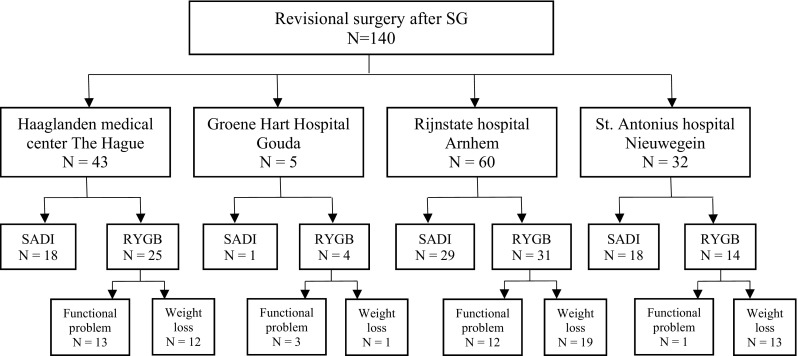
Number of patients included per clinic and division for type of surgery; SADI single anastomosis duodenoileal bypass, RYGB Roux-en-Y gastric bypass, SG sleeve gastrectomy

All collected data were analyzed retrospectively. Normally distributed values were presented as mean ± standard deviation and non-normally distributed as median with range. Chi-square tests were used to compare complication rates and the presence of vitamin and mineral deficiencies. Weight loss was only compared for patients with weight improvement as a main indication for surgery. Linear regression analysis was performed to compare %TBWL at 6, 12, and 24 months post-surgery for the SADI and RYGB. Missing data for weight loss at these time points was solved with linear interpolation imputation by taking the average of the value before and after the missing time point. Furthermore, linear mixed models was used to analyze the progression of %TBWL. As potential confounders that might be associated with both weight loss and the type of surgery performed, an analysis with the following variables was performed: gender, age at secondary surgery (date of secondary surgery—date of birth), center, pre-operative weight (measured the day of surgery or the day before), and minimum weight post-SG (lowest weight obtained before secondary surgery). On a theoretical basis, no variables were considered to be potential effect modifiers. *P* values of < 0.05 will be considered as statistically significant. All statistical analyses were run with the IBM SPSS Statistics version 24.0 for windows (Fig. 1).

## Results

One hundred forty morbidly obese patients underwent revisional surgery after primary SG. A SADI was performed on 66 patients and 74 patients were converted to a RYGB. All of the SADI patients were operated to improve weight loss. Indications for revision to a RYGB were insufficient weight loss in 39 (52.7%) patients, SG-related functional problems or reflux in 29 patients (39.1%), or a combination of both in 6 patients (8.1%). An overview of the baseline characteristics is given in Table 1. The SADI group had a higher average pre-operative BMI, was younger, had a shorter hospital stay, and underwent surgery later after a SG compared to the RYGB group.

**Table 1:** Baseline characteristics and operation related variables

	SG		SADI		RYGB		*P* value
	*N* = 140		*N* = 66		*N* = 74		
Age in years	41.3	(± 11.1)	43.3	(± 11.0)	45.1	(± 10.9)	.344
Sex ratio (F:M)	114:26		55:10		59:16		.367
Weight, kg	154.4	(± 30.6)	130.3	(± 22.0)	113.1	(± 25.3)	< .001
BMI, kg/m2	53.9	(± 10.3)	45.6	(± 6.9)	39.3	(± 7.9)	< .001
Minimum weight post-sleeve, kg			124.8	(± 24.5)	97.1	(± 22.3)	< .001
Years after sleeve			3.1	(1.0–14.9)	2.1	(0.3–6.8)	.001
Operative time, minutes	71.6	(± 23.6)	84	(40–199)	78	(39–212)	.869
Hospital stay after surgery, days			1	(1–8)	2	(1–25)	.002
Comorbidities (%)							
Hypertension			46.7%		49.3%		.814
Diabetes mellitus			20.0%		32.4%		.238
Dyslipidemia			21.4%		37.8%		.282
OSAS			20.6%		11.6%		.167

*P* values indicate differences between SADI and RYGB; *SD* standard deviation, *BMI* body mass index, *SG* sleeve gastrectomy, *SADI* single anastomosis duodenoileal bypass, *RYGB* Roux-en-Y gastric bypass, OSAS obstructive sleep apnea syndrome

Outcomes given in number with standard deviation or median with range

### Confounders

As potential confounders that might be associated with both weight loss and the type of surgery performed, the following possible confounding parameters were analyzed using mixed models: gender, age at secondary surgery, center, pre-operative weight, and minimum weight post-SG. Of these variables, none managed to change the difference in %TBWL between RYGB and SADI with more than 10%. Therefore, no confounding factor was apparent in this study.

### Weight Loss

**Fig. 2:**
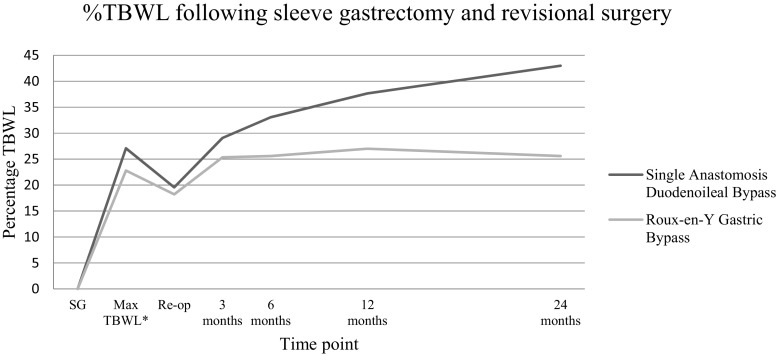
Average percentage total body weight loss after sleeve gastrectomy (SG) and revision to single anastomosis duodenoileal (SADI) bypass or Roux-en-Y gastric bypass (RYGB); SG sleeve gastrectomy, *Maximum %TBWL obtained after sleeve gastrectomy and before revisional surgery

Weight loss was analyzed only for patients with insufficient weight loss or weight regain as a main indication for surgery (SADI *n* = 66, RYGB *n* = 45). Before revisional surgery, mean BMI for these patients was 45.6 (± 6.9) kg/m^2^ in the SADI group and 42.5 (± 6.0) kg/m^2^ in the RYGB group. Mean BMI 2 years after secondary surgery was 32.7 (± 7.0) kg/m^2^ for the SADI group and 39.5 (± 5.5) kg/m^2^ for the RYGB group. To adjust for the difference in weight between the groups at baseline, the %TBWL was calculated. Firstly, the progression of %TBWL after revisional surgery is shown in Table 2, with corresponding *P* values for the differences between SADI and RYGB. An overview of the %TBWL over time is given in Fig. 2, calculated with the weight prior to SG as baseline. Secondly, the average %TBWL was calculated with mixed models. It was found that the RYGB had an average %TBWL of 6.3% and SADI of 16.5% over time, leading to a difference of 10.2% in favor of SADI patients (*P* < .001). Weight loss results at 2 years following revisional surgery were available for 47% (9/21) of SADI patients and 52% (22/42) of RYGB patients.

**Table 2:** Percentage total body weight loss (%TBWL) following secondary surgery

	%TBWL at 3 months	%TBWL at 6 months	%TBWL at 12 months	%TBWL at 24 months
SADI	11.3% (± 4.1)	16.5% (± 5.8)	21.5% (± 8.1)	26.4% (± 10.4)
RYGB	5.9% (± 5.3)	7.8% (± 6.8)	8.9% (± 8.7)	6.9% (± 11.3)
*P* value	< .001	< .001	< .001	< .001

*SADI* single anastomosis duodenoileal bypass, *RYGB* Roux-en-Y gastric bypass, *±* standard deviation in percentage

### Complications

An overview of complications is given in Table 3. Reasons for readmission to the hospital were abdominal pain, high fever, or persistent nausea. No peri- or post-operative mortality was observed in both groups. Within the first year of surgery, 11 (16.7%) complications were observed after a SADI and 13 (17.6%) after a RYGB (*P* = .888). Choledocholithiasis for which a laparoscopic cholecystectomy was performed (*n* = 7) was not counted as a complication. This occurred in two patients following SADI and five after RYGB. Furthermore, two of the medium-term complications in the SADI group included reoperation in the form of a re-sleeve because of insufficient weight loss. One patient presented with severe chronic diarrhea after SADI and underwent a subsequent duodenojejunostomy at 150 cm from the ligament of Treitz for enteral feeding.

**Table 3:** Short-term (< 30 days) and medium-term (> 1 month and < 12 months) complications

	SADI	RYGB	Total	*P*
	*N* = 66 (%)	*N* = 74 (%)	*N* = 140 (%)	
Short-term complication (< 30 days)	4 (6.1%)	6 (8.1%)	10 (7.1%)	.639
Readmission	3	4	7	
Reoperation	1	2	3	
Abscess	1			
Anastomic leakage		1		
No focus		1		
Med-term complication (> 1 and < 12 months)	7 (10.6%)	7 (9.5%)	14 (10%)	.821
Readmission	1	3	4	
Reoperation	6	4	10	
Internal herniation		2		
Incisional hernia	1			
Anastomic leakage	1			
Revisional surgery*	1			
Re-sleeve	2			
Stenosis		1		
No focus	1	1		

*SADI* single anastomosis duodenoileal bypass, *RYGB* Roux-en-Y gastric bypass

*Duodenojejunostomy at 150 cm from the ligament of Treitz for enternal feeding

### Nutritional Status

Although patients were advised to use specialized multivitamins, a deficiency was found in 30 (64%) SADI patients and in 28 (62%) RYGB patients (*P* = .705), during the 2-year follow-up. The absolute number of deficiencies with corresponding percentages can be found in Table 4. The data that was available per nutrient varied from 30 to 71%.

**Table 4:** Post-operative nutritional deficiencies within the first 2 years after revisional SADI and RYGB

	Post-SADI	Post-RYGB	
	*N* = 20–47*	*N* = 29–42*	
	Number of deficiencies (%)	Number of deficiencies (%)	*P* value
Anemia	16 (34%)	11 (26%)	.421
Ferritin	6 (14%)	11 (31%)	.071
Folate	10 (31%)	5 (12%)	.066
Vitamin B12	0	13 (33%)	< .001
Vitamin D	13 (28%)	9 (23%)	.587
Parathyroid hormone	3 (7%)	3 (8%)	.875
Calcium	3 (7%)	2 (5%)	.705
Albumin	5 (12%)	5 (17%)	.525
Vitamin B1	1 (5%)	0	N.A.
Vitamin B6	0	0	N.A.

*SADI* single anastomosis duodenoileal bypass, *RYGB* Roux-en-Y gastric bypass

*Dependent on nutritional value

### Functional Problems and GERD after SG

Thirty-five patients presented with functional problems after SG. Eight patients were diagnosed with therapy resistant GERD, 20 patients experienced dysphagia (for example due to a stenosis), five patients had persistent complaints of nausea and vomiting, and one presented with a fistula. A conversion to RYGB was performed in all of these patients. Cases of dysphagia or fistulas were all solved after revisional surgery to a RYGB. For patients with GERD, complaints remarkably improved for all patients. However, two out of eight patients still had GERD-related symptoms occasionally.

## Discussion

The present study investigated the effectiveness of the single anastomosis duodenoileal (SADI) bypass versus the Roux-en-Y gastric bypass (RYGB) on health outcomes as a revisional procedure after a failed sleeve gastrectomy (SG). To our knowledge, this study, which included 66 SADI and 74 RYGB patients, is the first to compare the SADI and RYGB as a second step operation for insufficient weight loss or weight regain.

The main results of the current study demonstrate good definitive weight loss results following SADI, with a %TBWL of 26% and mean BMI of 33 kg/m2 at 24 months following revisional surgery. Compared to the RYGB, the SADI resulted in significantly better weight loss (*P* = <.001). Moreover, 72% of RYGB patients regained a part of their lost weight 2 years after revisional surgery; opposed to SADI patients, who seem to progressively lose weight during the 2-year follow-up period. Another important finding was the comparable rate of complications following SADI and RYGB in the first year following surgery. Furthermore, a similar amount of deficiencies was observed between the two procedures.

Two previous studies evaluated weight loss after a SADI procedure with a prior SG and found excellent results, with a percentage excess weight loss of 70–80% after 24 months [11, 13]. These findings are similar to our results, where a percentage excess weight loss of 78% was found, and adds evidence to the notion that a SADI after SG provides consistent weight loss, even in different populations. When the RYGB after SG is compared to the existing literature, there seems to be an agreement on a peak in weight loss after 12 months and a decline in weight loss or even regain after this period [26, 27]. The overall level of %TBWL reported in these papers seems to be slightly higher than those obtained in our study but does not exceed 20%. Another proposed procedure to improve weight loss after a prior SG is the revisional sleeve gastrectomy (Re-SG), which is particularly promising when the original sleeve has dilated. It is found that the overall percentage of excess weight loss following the Re-SG can reach up to 57% at 12 months and up to 60% at 20 months follow-up [28, 29]. These percentages seem to exceed weight loss reported for RYGB in the current study; however, contrary to these outcomes Alsabah, S. et al. found more weight loss following revisional RYGB than after a Re-SG [29]. Still, they are not as high as those obtained after a SADI.

A second outcome evaluated in the present study was the rate of complications after revisional surgery. The SADI was originally developed as a modification of the biliopancreatic diversion with duodenal switch (BPD-DS). A reduction to just one anastomosis in SADI might be the reason why there are less complications when compared to the BPD-DS. Few studies compared the BPD-DS with RYGB after SG and found a generally higher amount of complications following BPD-DS; however, not significant [30, 31]. The present study found a similar complication rate; however, it can only be speculated on whether the SADI is an actual improvement for complication rates due to scarcity of this topic in the existing literature.

In studies comparing nutritional deficiencies after SADI, generally a higher percentage of deficiencies is described than observed in our patients [11, 13]. A difference in common channel length might have played a role in this, as previous studies included some cases with a common channel length of 200 cm, whereas the length was 250 cm for all but four of our patients. The authors mentioned that patients with a shorter common channel length were also more likely to develop deficiencies. Homan, J. et al. (2015) compared nutritional values for RYGB and BPD-DS after SG and found more deficiencies after BPD-DS (82% vs. 57%); however, not significant due to a small sample size [30]. The observation of a similar amount of nutritional deficiencies found in our sample provides some support for the hypothesis that deficiencies are less common after a SADI than after the traditionally performed BPD-DS.

The underlying mechanism for the difference in weight loss between the SADI and RYGB after SG might be explained by the difference in common channel and biliopancreatic limb length. It is assumed that a common channel length of 250 cm (with the exception of four common channels of 300 cm) in combination with a longer biliopancreatic limb length in SADI increases the malabsorptive component when compared to the RYGB leading to far better weight loss. Besides, a decline in weight loss or stabilization in weight 1 year after RYGB surgery is shared by several studies and can be considered as a failure of the procedure [32, 33]. Therefore, it may be questioned whether the RYGB should still be considered as an option for patients seeking to improve their weight loss after SG. Yet, many surgeons and patients are reluctant to choose a more invasive malabsorptive procedure such as the duodenal switch or SADI because of the disadvantages in terms of complications and deficiencies. It is argued that these disadvantages outweigh the benefits of more weight loss following these procedures when compared to the RYGB [8]. Perhaps these arguments might have led to a form of selection bias, in which patients with a higher pre-operative BMI are more likely to undergo a SADI, as can be seen in our data by the difference in baseline BMI before revisional surgery. However, after evaluation of the current results, it is reasonable to conclude that the disadvantages have been overemphasized, as was also mentioned previously by Sánchez-Pernaute, A. et al. [11]. In addition, identical operative times were observed and SADI patients experienced a shorter hospital stay after surgery. However, it should be noted that patients who presented with a functional problem are advised to undergo a RYGB. These patients were perhaps more prone to complications, leading to a higher rate of complications and a longer hospital stay following a RYGB.

Another concern associated with bariatric procedures that have larger malabsorptive characteristics, such as a SADI, is an increase in the occurrence of nutritional deficiencies. In the present study, the similarity of post-operative deficiencies found in both groups is likely related to sufficient supplementation, as every patient is advised to take specialized multivitamins to meet their daily requisite of vitamins after surgery and to prevent nutritional deficiencies from occurring. Moreover, patients are under strict guidance to meet their daily protein intake and multiple laboratory check-ups in the first year and yearly after that should ensure early detection of deficiencies and suitable treatment. As such, compliance to a strict vitamin regime is mandatory. It has been previously addressed that the super-morbid obese population is characterized for their extreme non-compliance [34], which might negatively impact the issue. This finding emphasizes the importance of regular follow-up for blood tests and a strict program by the clinic in which patients are treated.

A RYGB can be performed as revisional surgery if patients present with functional problems after a SG, such as dysphagia due to a stenosis or GERD. A narrow sleeve can be the cause of both of these problems; therefore, a SADI will most likely not solve the problem as the sleeve is left untouched. When a SG is converted into a RYGB, the sleeve is dissected to form a gastric pouch with a Roux-en-Y construction. As a result, problems attributed to a previous sleeve should resolve by bypassing the pylorus and promoting gastric emptying. In the present study, all patients but two were free of complaints after RYGB as a revisional procedure.

We acknowledge that our study has several limitations. Firstly, this includes the retrospective nature of our study, despite of the prospectively collected data in medical records. Secondly, an important factor after bariatric surgery which was not taken into account in the present study because of missing data is quality of life. Even though the SADI group did lose more weight, this does not necessarily lead to a better quality of life. Finally, as mentioned before, the super-morbid obese patients are known for their non-compliance. This was noticeable in our data by the significant amount of missing data. However, because of the frequent follow-up protocol, longitudinal data was mostly available. Points that contributed to the strength of our study were the relatively high number of patients included when compared to previous research. Furthermore, the present study combined results of multiple centers, which improves external validity of the results. Additionally, the SADI procedure as a second step has not been around for long and is still viewed as experimental; therefore, results that yield up to and including two-year follow-up are warranted [35].

## Conclusion

In conclusion, revisional surgery following SG into RYGB or SADI are both feasible options, with a similar risk for complications and nutritional deficiencies. For cases of GERD or functional problems after SG, a conversion to RYGB is preferred. However, conversion into a SADI offers significantly more weight loss without increased short- and long-term morbidity and may therefore be considered the recommended operation for patients seeking weight improvement.
